# Supervised Machine Learning and Physics Machine Learning approach for prediction of peak temperature distribution in Additive Friction Stir Deposition of Aluminium Alloy

**DOI:** 10.1371/journal.pone.0309751

**Published:** 2025-04-04

**Authors:** Akshansh Mishra, Vijaykumar Jatt, Eyob Messele Sefene, Sachin Salunkhe, Robert Cep, Emad Abouel Nasr

**Affiliations:** 1 School of Industrial and Information Engineering, Politecnico Di Milano, Milan, Italy; 2 Department of Mechanical Engineering, School of Engineering and Applied Sciences, Bennett University, Noida, Uttar Pradesh, India; 3 Department of Mechanical Engineering, National Taiwan University of Science and Technology, Taipei, Taiwan; 4 Department of Biosciences, Saveetha School of Engineering, Saveetha Institute of Medical and Technical Sciences, Chennai, Tamil Nadu, India; 5 Department of Mechanical Engineering, Gazi University Faculty of Engineering, Maltepe, Ankara, Turkey; 6 Department of Machining, Assembly and Engineering Metrology, Faculty of Mechanical Engineering, VSB-Technical University of Ostrava, Ostrava, Czech Republic; 7 Department of Industrial Engineering, College of Engineering, King Saud University, Riyadh, Saudi Arabia; Xi’an Jiaotong University, CHINA

## Abstract

Additive friction stir deposition (AFSD) is a novel solid-state additive manufacturing technique that circumvents issues of porosity, cracking, and properties anisotropy that plague traditional powder bed fusion and directed energy deposition approaches. However, correlations between process parameters, thermal profiles, and resulting microstructure in AFSD still need to be better understood. This hinders process optimization for properties. This work employs a framework combining supervised machine learning (SML) and physics-informed neural networks (PINNs) to predict peak temperature distribution in AFSD from process parameters. Eight regression algorithms were implemented for SML modeling, while four PINNs leveraged governing equations for transport, wave propagation, heat transfer, and quantum mechanics. Across multiple statistical measures, ensemble techniques like gradient boosting proved superior for SML, with the lowest MSE of 165.78. The integrated ML approach was also applied to classify deposition quality from process factors, with logistic regression delivering robust accuracy. By fusing data-driven learning and fundamental physics, this dual methodology provides comprehensive insights into tailoring microstructure through thermal management in AFSD. The work demonstrates the power of bridging statistical and physics-based modeling for elucidating AM process-property relationships.

## 1. Introduction

Additive manufacturing (AM) has revolutionized production across diverse industries by directly enabling on-demand fabrication of complex geometries from digital models [1–5]. While powder-bed fusion and directed energy deposition techniques dominate the metal AM landscape, they inherently suffer from issues related to the melt-pool mode of material addition and subsequent rapid solidification. Porosity, cracking, residual stresses, and anisotropic properties are typical [6–9]. Solid-state metal AM approaches have emerged in recent years, seeking to circumvent these challenges by avoiding bulk melting of feed material. Additive Friction Stir Deposition (AFSD) is a novel technique combining friction stir processing concepts and additive layer manufacturing. First proposed in 2018, it has garnered significant interest for promising superior microstructure and properties compared to other metal AM methods [10–15].

The primary benefit of AFSD is its ability to produce fully dense parts with properties comparable to those of wrought alloys. This is particularly important in aerospace, automotive, and defense industries, where high structural integrity and mechanical performance are crucial [16–19]. In addition, AFSD allows for localized deposition, enabling the precise replacement of lost material while maintaining the original properties of the component. This makes it an ideal solution for repairing and refurbishing high-value components damaged due to wear, corrosion, or impact. Another significant advantage of AFSD is its capability for solid-state recycling of machining chips and scraps. By converting these materials into usable metal powder feedstock, AFSD enables sustainable in-house recycling, reducing waste and minimizing environmental impact. Moreover, AFSD offers higher deposition rates than other additive manufacturing techniques, such as powder bed fusion and directed energy deposition, allowing for the rapid production of large parts. The unique deposition mechanism employed by AFSD also produces parts with improved microstructural properties. The as-deposited microstructure is characterized by fine-grained, equiaxed grains resulting from dynamic recrystallization during deposition. These grains lead to isotropic properties superior to the coarse columnar grains found in other AM processes. Furthermore, the solid-state deposition process used in AFSD generates compressive residual stresses that enhance fatigue life and damage tolerance, whereas other AM methods typically induce tensile residual stresses. The peak temperature distribution significantly influences microstructure evolution during AFSD. A comprehensive understanding of this relationship is necessary to predict and control microstructural features, such as grain size and shape, which directly impact the mechanical properties of the deposited material. High peak temperatures can generate residual stresses that may result in warpage or distortion of the workpiece [20–22]. By examining the correlation between peak temperature distribution and residual stress/distortion, this study aims to provide guidelines for mitigating these issues and ensuring dimensional accuracy. During AFSD can profoundly affect the material’s properties, including its microstructure, hardness, and corrosion resistance. Analyzing the peak temperature distribution can help researchers and manufacturers better understand its formation and develop strategies to minimize any detrimental effects. Uncontrolled peak temperatures can lead to thermal damage, alter the material’s chemical composition, promote phase transformations, or even cause burnout. By monitoring peak temperature distribution, this study seeks to identify threshold values that can prevent these unwanted phenomena and ensure the quality of the deposit. Different materials exhibit unique responses to the same processing conditions. Investigating peak temperature distribution across diverse materials can reveal material-specific trends and guide the development of tailored AFSD processes that account for these differences. Peak temperature distribution is crucial in determining the optimal process parameters for achieving desired microstructures and mechanical properties. By analyzing the effects of processing conditions on peak temperature distribution, this study seeks to establish correlations to optimize AFSD processes.

While additive manufacturing techniques like powder bed fusion and directed energy deposition are commonplace, they need help with issues like porosity, cracking, and poor mechanical properties due to the melt-pool mode of material addition. Additive friction stir deposition (AFSD) has recently emerged as a novel solid-state technique combining friction stir processing and layer-wise deposition to circumvent these challenges. However, a comprehensive understanding of process-property-microstructure relationships in AFSD must be improved. In particular, the correlation between peak temperature distribution and resulting microstructural evolution during AFSD still needs to be better understood. Prior work has yet to systematically analyze peak temperature profiles in AFSD to identify process-microstructure linkages. This knowledge gap hinders the optimization of AFSD processes for desired properties.

This research addresses this gap by employing a dual supervised and physics-informed machine learning approach to predict peak temperature distribution from AFSD process parameters. By fusing data-driven learning and fundamental process physics, this work uniquely combines the strengths of both techniques to enhance model accuracy. The integrated framework is applied to modeling thermal phenomena in metal additive manufacturing. Outcomes from this data-driven and physics-based modeling will provide novel, clinically relevant insights into tailoring microstructural features like grain morphology and size distribution through controlled thermal management. By clarifying peak temperature-property correlations, this work will facilitate the design of optimized AFSD processes for properties like strength and damage tolerance.

## 2. Comparison of supervised ML and physics-based ML

**Fig 1:**
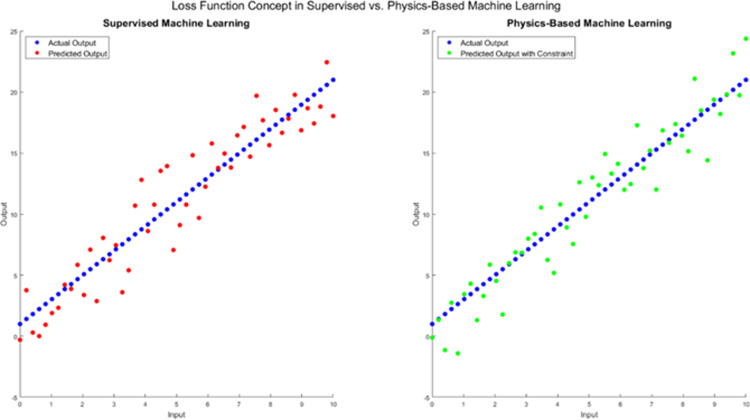
Concept of the Loss function in the case of Supervised Machine Learning and Physics Machine Learning approach.

Supervised and physics-based machine learning are two approaches to building machine learning models. Training a model with labeled data in supervised machine learning entails knowing the target outcome for each input. For the model to make predictions on fresh, unforeseen data, it must be learned a mapping from inputs to outputs. The model’s parameters are changed to reduce this loss after training using a loss function to measure the discrepancy between expected and actual results. Image classification, audio recognition, and sentiment analysis are supervised machine learning tasks. On the other hand, physics-based machine learning entails including physical rules or restrictions in the machine learning model. This strategy is beneficial when working with complicated systems whose basic physical principles are clear but whose behavior is unpredictable because of the sheer volume of data involved. Physics-based machine learning models forecast the behavior of such systems and ensure that the predictions align with the underlying physical rules. The discrepancy between the expected and actual output is frequently used to determine the loss function in supervised machine learning. The loss function in physics-based machine learning may contain terms that require fulfilling physical restrictions or rules, such as energy conservation or momentum, as shown in Fig 1. While physics-based machine learning can work with unlabeled data and use the physical laws as a guide to discover the patterns in the data, supervised machine learning requires tagged training data. While physics-based machine learning models are created with the physical laws incorporated into the model architecture, supervised machine learning models are frequently created without prior knowledge of the underlying physical principles. Unlike physics-based machine learning models, which may make probabilistic predictions that account for measurement noise and uncertainty in the physical laws, supervised machine learning models only make point predictions. Physics-based machine learning models are frequently easier to interpret than supervised machine learning models because they shed light on the fundamental principles that underlie the system’s behavior. In contrast to supervised machine learning models, physics-based machine learning models can be relatively cheaper to train and evaluate computationally, especially when dealing with complicated physical systems.

## 3. Materials and methods

**Fig 2:**
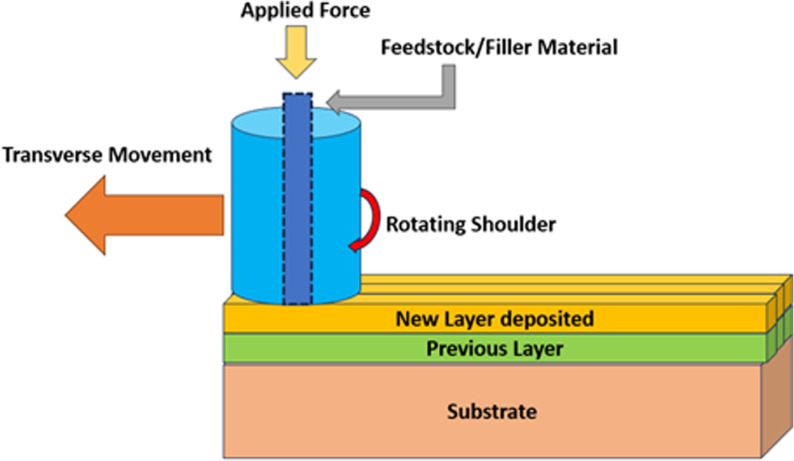
Schematic representation of the Additive Friction Stir Deposition process.

Additive Friction Stir Deposition (AFSD) is a solid-state additive manufacturing process that does not involve melting the deposited material. It is based on the principles of friction stir welding and combines concepts of material deformation processing and layer-by-layer additive fabrication, as shown in Fig 2. In AFSD, the material to be deposited is supplied as a rod or powder into the rotating non-consumable tool. As the tool contacts the substrate or previous layer, friction at the interface generates heat, which softens the feed material, allowing it to deform plastically. For powder feedstocks, additional external heating may be required for proper consolidation. The rotating action of the tool provides mixing and consolidation of the feed material, which extrudes under pressure to fill the gap between the tool and substrate.

**Fig 3:**
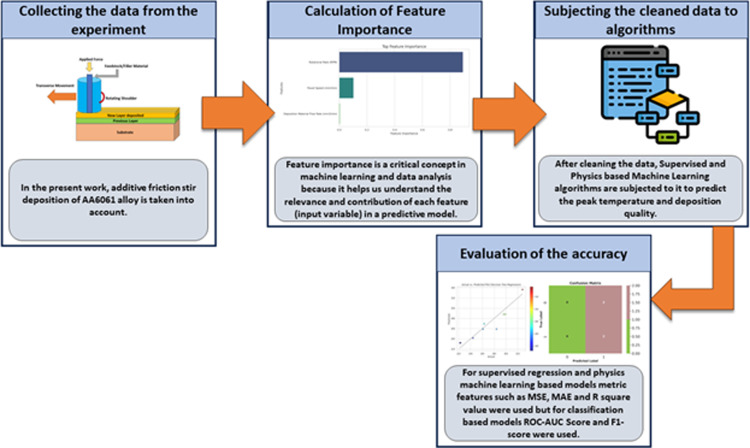
The machine learning framework implemented in the present work.

The severe plastic deformation at elevated temperatures leads to metallurgical bonding between the deformed feed material and substrate material through interdiffusion at the interface. This bonding occurs entirely in the solid state, unlike in melt-based processes. As the tool traverses the substrate, it deposits a track of the solid-state deformed material. By repeating this process in a layer-by-layer fashion, complex 3D metal parts can be fabricated. The unique thermomechanical conditions of AFSD lead to dynamic recrystallization of the deposited material, resulting in a fine equiaxed grain structure. This is unlike the coarse columnar grains commonly observed in melt-based additive manufacturing processes, which suffer from problems like porosity, cracking, and anisotropic properties. The fine-grained microstructure achieved in AFSD enhances the material properties. The main framework implemented in the present work is shown in Fig 3. The data [23,24] were prepared and further imported to the Google Colab platform for subjecting it to machine learning algorithms coded using Python. Data preprocessing techniques: Outlier Detection and Removal: Outliers can significantly affect the performance of machine learning models. Visualization method namely scatter plot used to detect and handle outliers. Feature Selection: Statistical test namely F-test used to select the most relevant features. The initial input parameters considered in the present work are Rotational Rate (RPM), Travel Speed (mm/min), Tool Geometry, Deposition Material Flow Rate (mm^3^/min), Tool Diameter (mm), and Powder Size (micrometer). In contrast, the output parameters are Peak temperature (degree Celsius) and the deposition quality. The data were further divided into a training set and a testing set, i.e., 80 percent of the data were used for training and 20 percent for testing purposes. The data were subjected to eight supervised machine learning regression-based algorithms and four physics-based machine learning algorithms to predict the peak temperature. The data were subjected to nine classification-based machine learning algorithms to predict the deposition quality. In order to evaluate the performance of supervised regression and physics machine learning-based models, metric features such as MSE, MAE, and R square value were used. However, for classification-based models, the ROC-AUC Score and F1-score were used.

## 4. Results and discussion

### 4.1. Supervised machine learning regression algorithms for peak temperature prediction

**Fig 4:**
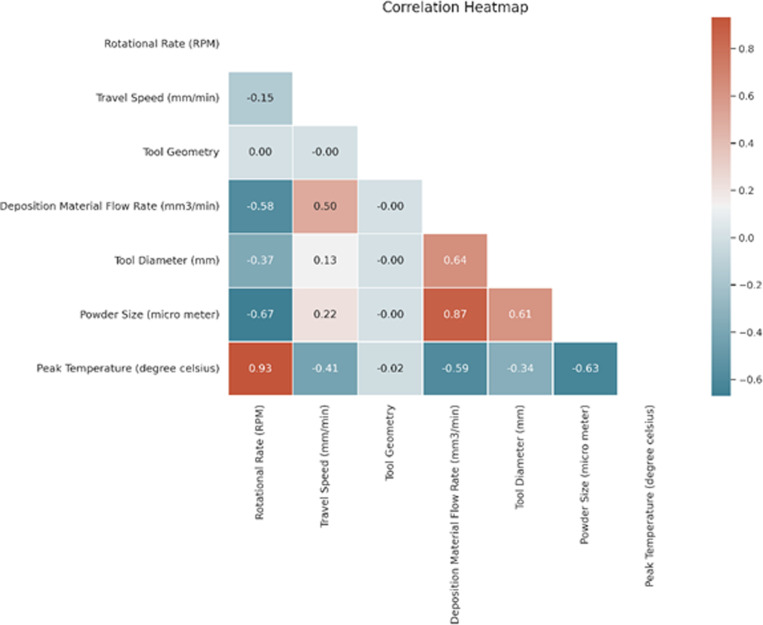
Obtained heat map for regression-based algorithms.

Fig 4 shows the correlation heatmap obtained for supervised machine learning regression-based algorithms. The strong positive correlation between Rotational Rate (RPM) and Peak Temperature. There is also a moderate positive correlation between Travel Speed and Peak Temperature. This indicates that RPM and Travel Speed are essential predictors of Peak Temperature.

**Fig 5:**
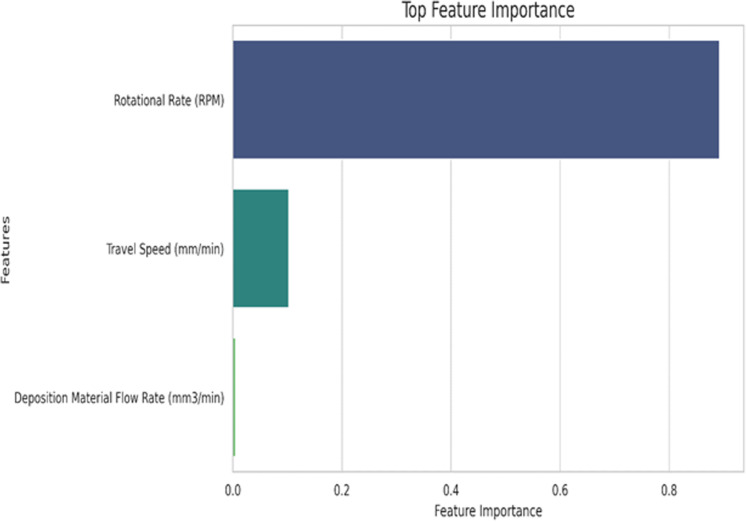
Feature importance plot obtained for regression-based algorithms.


Fig 5 shows the feature importance plot; it is observed that the Rotational rate has the highest contribution towards the output parameter, i.e., towards the peak temperature value. Now, let us discuss the working mechanism and results from the implemented regression-based algorithms to predict the peak temperature. Support Vector Regression (SVR) is a powerful machine learning technique employed for regression tasks, particularly in cases where the relationships between input features and the output parameter are nonlinear and complex. In the context of the present research, there are three input parameters: Rotational Rate (RR), Travel Speed (TS), and Deposition Material Flow Rate (DMFR), denoted as xᵢ =  [RRᵢ, TSᵢ, DMFRᵢ]. The goal of SVR is to predict the output parameter, Peak Temperature (PT), represented as yᵢ. The primary mathematical objective of SVR can be expressed in Equation 1.

$$Minimize:\frac{1}{2}∥w{∥}^{2}+C.\sum_{i=1}^{n}\left[\varepsilon_{i}+\varepsilon_{i}^{*}\right]$$ (1)

where ‘w’ corresponds to the weight vector of the hyperplane, ‘C’ is the regularization parameter controlling the trade-off between maximizing the margin and minimizing the error, ‘ε_ᵢ_’ and ‘ε_ᵢ_*’ are slack variables representing the degree of violation of the margin by individual data points. The hyperplane is denoted by Equation 2.


$$f\left(x\right)=w^{T}\times x+b$$ (2)

where ‘f(x)’ signifies the predicted Peak Temperature (PT), ‘x’ is the input feature vector containing Rotational Rate (RR), Travel Speed (TS), and Deposition Material Flow Rate (DMFR), and ‘b’ is the bias term. SVR incorporates constraints to ensure that the predicted PT values align with the actual PT values within a specified margin (ε) shown in Equation 3.


$${\text{PT}}_{\text{i}}-\text{f}\left({\text{x}}_{\text{i}}\right)\leq {\text{ε + ε*}}_{\text{i}}\text{, f}\left({\text{x}}_{\text{i}}\right)-{\text{PT}}_{\text{i}}\leq {\text{ε+ε*}}_{\text{i}}$$ (3)

These constraints enforce the ability of SVR to handle errors within the margin while penalizing deviations exceeding this margin. These deviations are quantified using the epsilon-insensitive loss function shown in Equation 4.


$$L\left(\varepsilon ,\varepsilon *\right)=max\left(0,\left|PT_{i}-f\left(x_{i}\right)\right|-\varepsilon -\varepsilon {*}_{i}\right)$$ (4)

**Fig 6:**
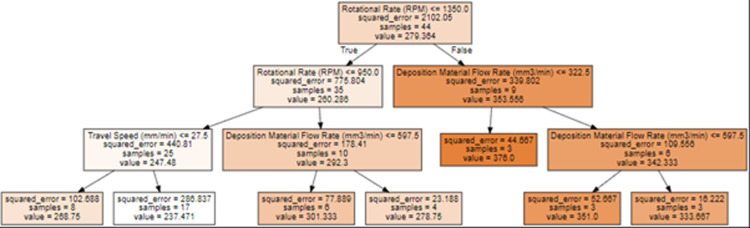
Decision Tree plot obtained in the present work.

This loss function incentivizes the SVR model to minimize errors while permitting those within the specified margin. The optimization task associated with SVR entails determining the optimal hyperplane coefficients ‘w’ and ‘b’ while respecting the constraints and minimizing the loss. When it comes to handling regression issues, Decision Tree Regression is a reliable and understandable machine-learning technique. This approach performs exceptionally well when the connections between the input characteristics and the output variable are intricate and nonlinear. The core mathematical idea of Decision Tree Regression is repeatedly dividing the dataset into subsets depending on the input features while striving to maximize a particular criterion. The forecast for a new input data point is created by averaging the training data points’ output values in the leaf node that the new data point belongs to. The algorithm explicitly chooses the feature that yields the best split at each tree node, often by reducing the mean squared error (MSE) shown in Equation 5.


$$MSE=\sum \frac{{\left(y_{i}-\bar{y}\right)}^{2}}{n}$$ (5)

where ‘yᵢ’ is the actual output value, ‘ȳ’ is the mean of the output values in the current node, and ‘n’ is the number of data points in the node. The feature and threshold that minimize the MSE are chosen to make the split. Decision Tree Regression repeats this process, adding additional nodes and splits until a stopping requirement, such as a maximum tree depth or a minimum amount of data points per leaf, is met. This produces a tree structure that maps input information to expected output values, as shown in Fig 6. The decision tree has a maximum depth of 3 levels and uses ‘Rotational Rate (RPM)’ as the feature for the root node split. This indicates that the Rotational Rate is the most important feature for predicting the target variable ‘Peak Temperature (degree Celsius)’. The tree uses Rotational Rate as the root node split, suggesting it is the most essential feature. For lower RPM ≤  1350, it further splits on Travel Speed. This shows that RPM and Travel Speed help segment the data into regions with different temperature means.

**Fig 7:**
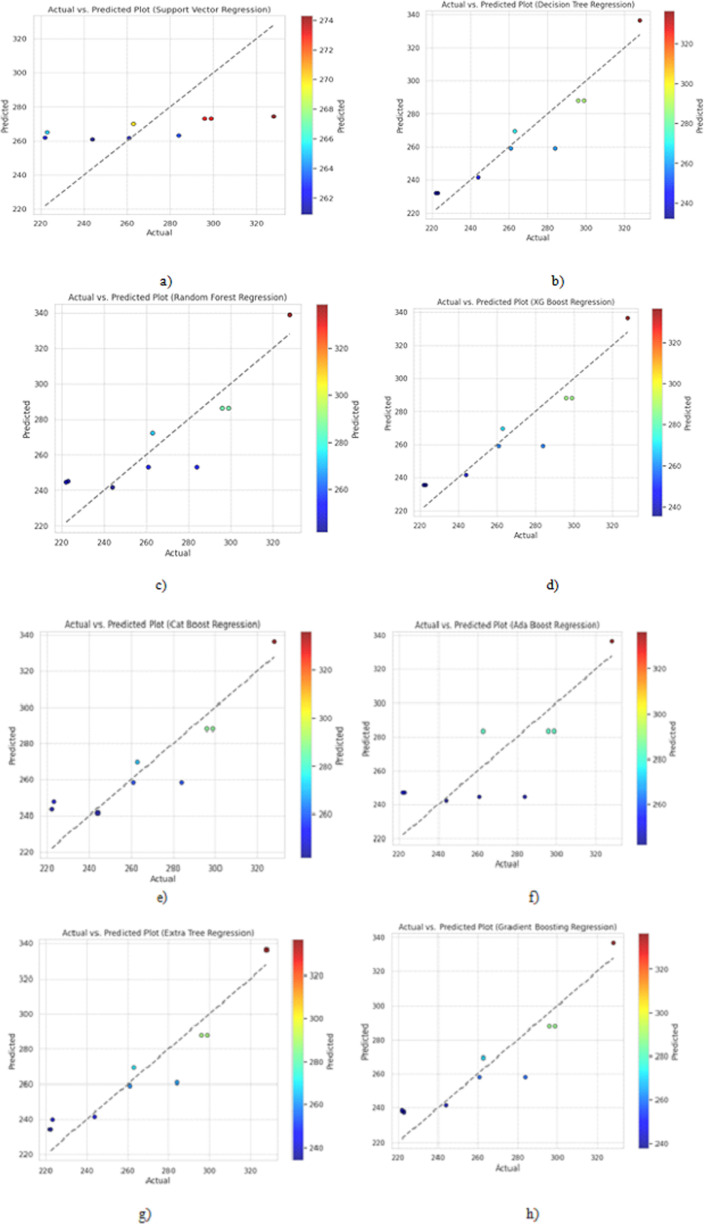
Actual vs Predicted value plots for a) Support Vector Regression, b) Decision Tree Regression, c) Random Forest Regression, d) XGBoost Regression, e) CatBoost Regression, f) AdaBoost Regression, g) Extra Tree Regression and h) Gradient Boosting Regression algorithms.

The Random Forest algorithm generates a set of decision trees in two steps: bootstrapped sampling and feature randomness. First, it creates numerous bootstrapped datasets by randomly selecting sections of the training data with replacements. Then, for each bootstrapped dataset, it builds a decision tree with only a random subset of the features considered at each node. The final prediction for a new input data point is derived by combining all the individual tree forecasts. This is typically accomplished by averaging the anticipated values for regression jobs. The Random Forest ensemble reduces overfitting in individual decision trees and increases overall forecast accuracy. It captures complex and nonlinear interactions between input and output parameters. The capacity to reduce variation while keeping low bias is critical to its effectiveness. Random Forest Regression is used in the present study to estimate Peak Temperature (PT) based on the input parameters Rotational Rate (RR), Travel Speed (TS), and Deposition Material Flow Rate (DMFR). The ensemble technique improves model generalization by providing robustness against noisy data. The Random Forest ensemble is a weighted average of individual decision trees, as shown in Equation 6.


$$f\left(x\right)=\frac{1}{N}\sum_{i=1}^{N}f_{i}\left(x\right)$$ (6)

where ‘f(x)’ represents the final prediction, ‘N’ is the number of trees in the ensemble, and ‘f_i_(x)’ is the prediction of the i^th^ decision tree. XGBoost (Extreme Gradient Boosting) is a cutting-edge ensemble learning algorithm that solves regression problems with fantastic accuracy and efficiency. This strong technique is based on gradient boosting principles, and it successively combines numerous weak predictive models (usually decision trees) into a robust and highly predictive ensemble. The core idea underlying XGBoost is to train decision trees iteratively to rectify errors generated by the ensemble of previously trained trees. This is accomplished by minimizing a specific loss function that quantifies the difference between expected and actual output values. To control model complexity, the approach combines regularization techniques and a sophisticated approximation strategy to speed up the optimization process. Furthermore, XGBoost includes features such as gradient boosting and boosting, a sophisticated ensemble strategy that iteratively updates the model using a weighted sum of the weak learners. XGBoost optimizes the objective function shown in Equation 7.


$$Obj=\sum_{i=1}^{n}L\left(y_{i},\hat{y_{i}}\right)+\sum_{k=1}^{K}\text{Ω}\left(f_{k}\right)$$ (7)

where ‘Obj’ is the overall objective, ‘n’ is the number of data points, ‘$L\left(y_{i},\hat{y_{i}}\right)$’ represents the loss function that quantifies the error for each data point, ‘K’ is the number of leaves in the tree, ‘$f_{k}$’ represents the prediction from the k-th tree, and ‘$\text{Ω}\left(f_{k}\right)$’ is a regularization term that penalizes complex models. CatBoost, short for Categorical boosting, is a cutting-edge gradient boosting technique that solves regression problems while effectively handling categorical variables. It is a huge step forward in ensemble learning and machine learning. CatBoost, like other gradient boosting systems, uses an ensemble of decision trees, but it adds numerous improvements that set it different. CatBoost handles categorical data natively without needing considerable preprocessing, as it leverages techniques like ordered boosting and oblivious trees. CatBoost also presents a novel way for dealing with overfitting using a per-leaf algorithm, effectively lowering model complexity. To train gradient-boosted ensemble models, the objective functions of XGBoost (Extreme Gradient Boosting) and CatBoost (Categorical Boosting) are similar in that they both strive to minimize a combination of a loss function and a regularization term. However, the particular formulations and specifics of the two algorithms may differ. XGBoost introduces regularization terms to regulate the complexity of the individual trees in the ensemble, preventing overfitting. On the leaf scores, these regularization terms include L1 (Lasso) and L2 (Ridge) regularization. CatBoost also employs a combined loss function and regularization goal function. CatBoost, on the other hand, offers particular approaches for dealing with categorical characteristics natively and addressing overfitting. AdaBoost, short for Adaptive Boosting, is a classification problem-solving ensemble learning technique. This approach is particularly good at integrating numerous weak classifiers to form a resilient and highly accurate ensemble classifier. AdaBoost’s primary strength is its capacity to adapt and provide greater weight to data points misclassified by the ensemble, allowing it to focus on complex situations while improving classification accuracy. AdaBoost’s main principle is to generate a robust classifier by iteratively merging the predictions of weak classifiers, which are frequently simple decision stumps (decision trees with a single split). Each weak classifier is trained on the dataset, with changed weights provided to the data points to highlight the misclassified ones. A weighted majority vote of these weak classifiers produces the final prediction. The weights of the ensemble’s weak classifiers are determined by their accuracy, with higher-accuracy classifiers having more effect. AdaBoost minimizes the exponential loss function shown in Equation 8.


$$L\left(f\right)=\sum_{i=1}^{n}e^{-y_{i}.f\left({}_{}\right)}\text{ix}$$ (8)

where ‘L(f)’ is the loss function, ‘n’ is the number of data points, ‘yᵢ’ is the actual label (+1 or -1), ‘f(x_ᵢ_)’ is the prediction of the classifier, and ‘e’ is the base of the natural logarithm. AdaBoost aims to find the weak classifiers ‘f_ᵢ_(x)’ and their corresponding weights ‘αᵢ’ that minimize this loss function. The Extra Trees Regressor, or Extremely Randomized Trees, is a regression-specific ensemble learning technique. This algorithm draws on the ideas of decision tree regression but adds randomness and variety to improve predictive performance. The Extra Trees Regressor is distinguished by its high degree of unpredictability during tree-building. Extra Trees generates numerous decision trees utilizing all available characteristics and selects random feature thresholds at each node, unlike typical decision trees, which find optimal splits based on a selection of features. It also introduces random data subsampling for training each tree. Extra Trees decreases the danger of overfitting while increasing ensemble diversity, resulting in a more robust and generalizable regression model. The Extra Trees Regressor uses a criterion such as mean squared error (MSE) or mean absolute error (MAE) to optimize for the optimal split at each node. A fresh input data point’s final prediction is derived by averaging the predictions of all the individual trees in the ensemble. At the same time, Gradient Boosting Regression is based on iteratively improving predictions by merging the outputs of numerous weak learners, often decision trees, into a robust ensemble model. A new weak learner is trained at each iteration to capture the errors or residuals of the current ensemble’s predictions. These learners are intended to rectify the faults of preceding ones, reducing the overall prediction error progressively. The final prediction for a new input data point is derived by adding the predictions from all individual trees and weighting them by a learning rate. The plots for Actual peak temperature values vs predicted peak temperature values are shown in Fig 7. Most models can fit the general increasing trend in the data. However, Support Vector Regression shows a poor fit with high variance. Decision Tree, XGBoost, CatBoost, and Gradient Boosting provide the best fit with less deviation from the actual values. This indicates that ensemble methods like gradient boosting perform well for this regression task.

**Fig 8:**
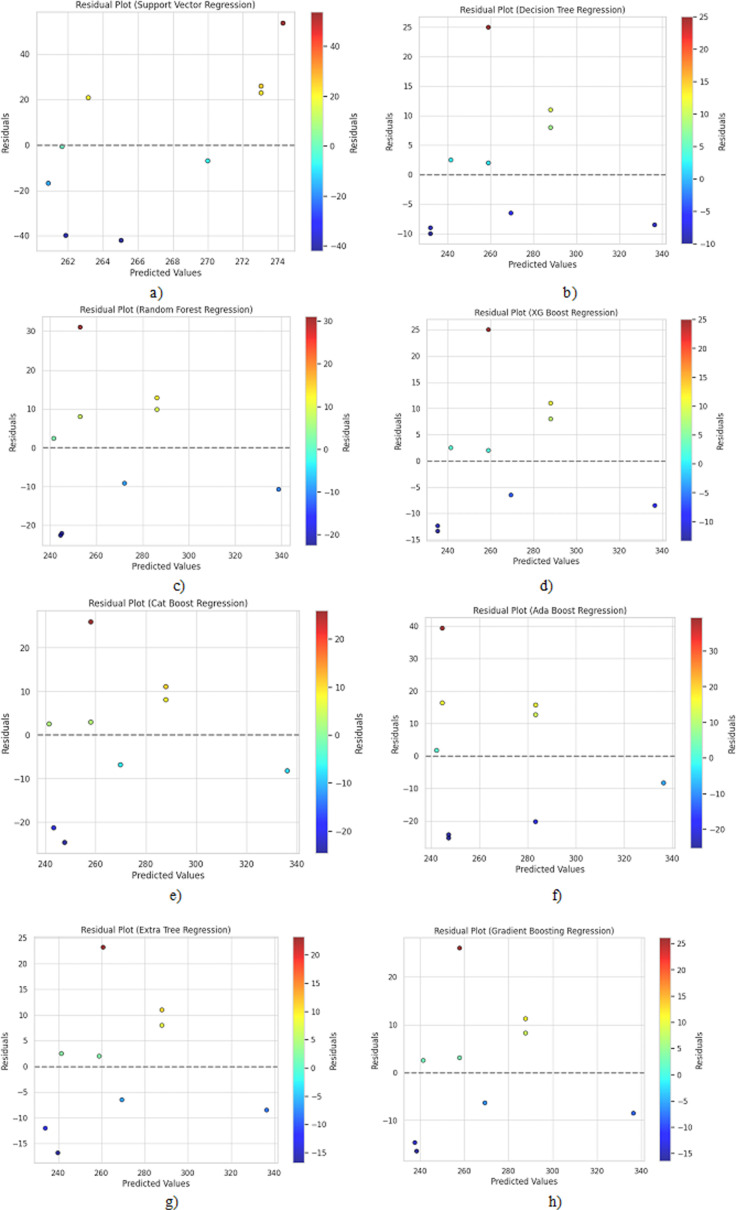
Residual plots for a) Support Vector Regression, b) Decision Tree Regression, c) Random Forest Regression, d) XGBoost Regression, e) CatBoost Regression, f) AdaBoost Regression, g) Extra Tree Regression and h) Gradient Boosting Regression algorithms.

Fig 8 shows the residual plots of the implemented regression-based algorithms in the present work. It is observed that the residuals (difference between actual and predicted values) are scattered randomly around 0 for most models, indicating no systematic pattern in the errors. However, Support Vector Regression shows a distinct funnel shape, suggesting more significant errors for extreme values.

**Fig 9:**
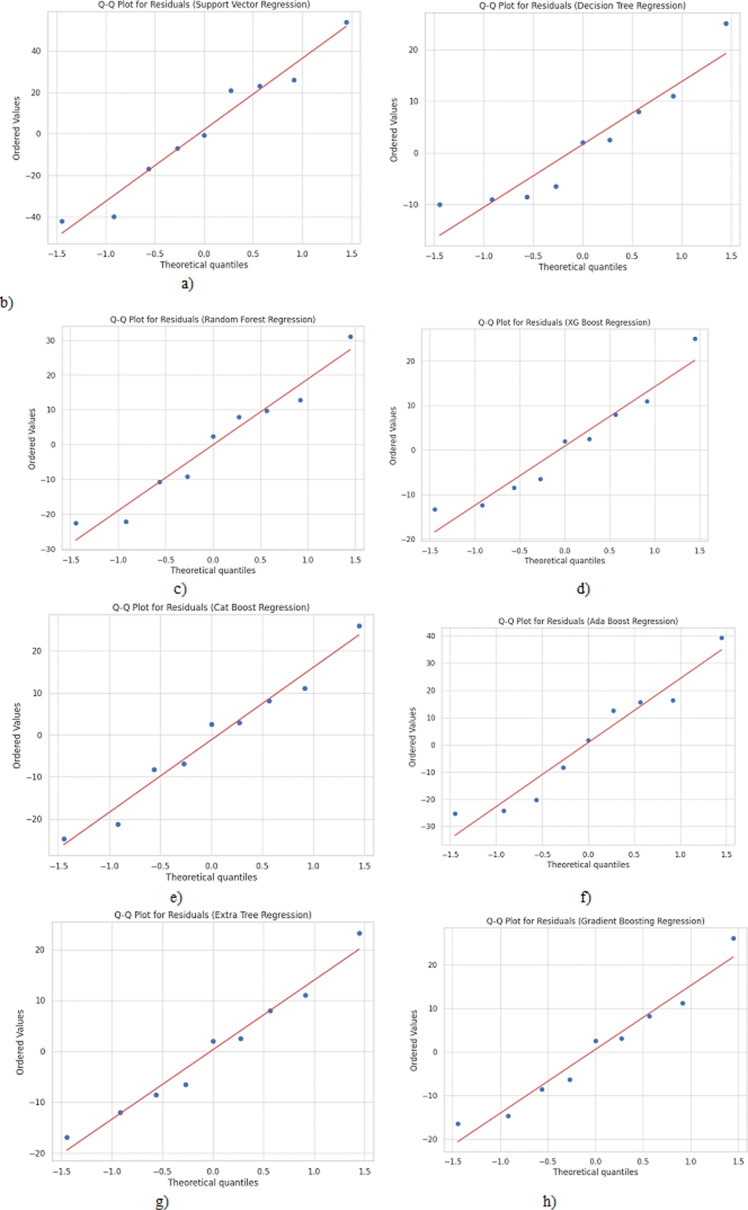
Q-Q plots for a) Support Vector Regression, b) Decision Tree Regression, c) Random Forest Regression, d) XGBoost Regression, e) CatBoost Regression, f) AdaBoost Regression, g) Extra Tree Regression and h) Gradient Boosting Regression algorithms.

Fig 9 displays Q-Q plots comparing the distribution of residuals to a normal distribution. The residuals should closely follow the regular line for a good model fit. Decision Tree, Random Forest, XGBoost, CatBoost, and Gradient Boosting residuals align well with normal distribution. Support Vector Regression shows significant deviations, reflecting poor fit.

Ensemble methods like gradient boosting, XGBoost, random forest, and CatBoost outperformed simpler regression algorithms like support vector regression for predicting peak temperature. This indicates that ensemble techniques that combine multiple weak learners are adequate for this task. From Table 1 it can be seen that, gradient boosting achieved the lowest MSE of 165.78 and a high R^2^ of 0.8563, demonstrating predictive solid performance. XGBoost and extra trees also produced low MSE values under 200. Support vector regression performed poorly, with a high MSE of 912 and a low R^2^ of 0.2095.

**Table 1:** Results obtained from the supervised machine learning regression-based algorithms in the present work.

Algorithms	MSE Value	MAE Value	R^2^ Value	Execution time in seconds
Support Vector Regression	912.0466	25.5410	0.2095	0.0170
Decision Tree Regression	123.9722	9.1666	0.8925	0.0155
Random Forest Regression	276.0956	14.2789	0.7607	0.2282
XGBoost Regression	140.7654	9.9169	0.8780	0.0828
CatBoost Regression	228.0816	12.3990	0.8023	1.0085
AdaBoost Regression	437.1336	18.2132	0.6211	0.1032
Extra Tree Regression	141.7946	10.0629	0.8771	0.0299
Gradient Boosting Regression	165.7845	10.8112	0.8563	0.1041

### 4.2. Physics-based machine learning algorithms for peak temperature prediction

This research also employed a Physics-Informed Neural Network (PINN) to predict Peak Temperature (PT) as a continuous output parameter based on three input parameters: Rotational Rate (RR), Travel Speed (TS), and Deposition Material Flow Rate (DMFR). The research problem can be framed mathematically as the PT (y) prediction as a function of the input features, xᵢ =  [RRᵢ, TSᵢ, DMFRᵢ]. The loss function, which combines two fundamental components, i.e., Physics-Informed Loss (PINN Loss) and Data-Driven Loss, is at the heart of the PINN model. The transport equation lies at the heart of this technique. It entails computing the derivatives of model predictions (u) concerning time (t) and position (x). The transfer equation is written as shown in Equation 9.


$$c.\frac{\partial u}{\partial t}+\frac{\partial u}{\partial x}=0$$ (9)

where ‘c’ represents the advection velocity. The PINN loss term, denoted as ‘pinn loss,’ ensures that the derivatives of the model predictions adhere to this transport equation, enforcing the physics-based constraints.

The data-driven component quantifies the error between the model’s predictions and target values. It is expressed as shown in Equation 10.


$$data\;loss=\frac{1}{n}\sum_{i=1}^{n}{\left(u_{i}-y_{i}\right)}^{2}$$ (10)

where ‘u_i_’ represents the model’s prediction for the i-th data point, ‘y_i_’ is the actual target value (Peak Temperature), and ‘n’ is the number of data points. The overall loss function, ‘loss,’ combines these two components, as shown in Equation 11.


loss=pinn loss+data loss (11)

During training, the PINN model minimizes this combined loss. Gradients are calculated using TensorFlow’s GradientTape, and gradient descent is applied using the Adam optimizer. The model learns to represent the complicated interactions between the input parameters (RR, TS, DMFR) and the output (PT) while adhering to the physics-driven limitations defined by the transport equation during the training phase. Following training, the model is used to predict on a separate testing dataset, and assessment measures such as Mean Squared Error (MSE) and Mean Absolute Error (MAE) are calculated to evaluate its predictive accuracy. Now let us discuss the Physics-based Machine Learning model based on the wave equation in terms of predicting the output parameter, Peak Temperature (PT). In predicting PT, the fundamental physics is represented by the wave equation shown in Equation 12. This equation captures the physics of wave behavior, indicating how the second derivatives of ‘u’ in both time and position are related.


$$c^{2}.\frac{\partial^{2}u}{\partial t^{2}}-\frac{\partial^{2}u}{\partial x^{2}}=0$$ (12)

where u’ represents the wave function (in this case, Peak Temperature, PT) and ‘c’ is the wave velocity, which characterizes how fast the wave propagates. The physics-informed loss term, ‘wave loss,’ assures that the model follows the wave equation as shown in Equation 13. The term ‘wave loss’ quantifies how well the model meets the physics-based wave equation, encouraging it to capture the physical behavior of the wave.


$$wave\;loss=\frac{1}{n}\sum_{i=1}^{n}\left({\left(c^{2}.\frac{\partial^{2}u}{\partial t^{2}}-\frac{\partial^{2}u}{\partial x^{2}}\right)}^{2}\right)$$ (13)

The data-driven loss is calculated using Equation 10, and the overall loss is calculated using Equation 14.


loss=wave loss+data loss (14)

This complete loss metric directs the PINN model’s training process, allowing it to provide accurate Peak Temperature predictions while adhering to the physics-based wave equation and efficiently fitting the given data. In the case of the physics-based machine learning model based on the heat equation, the prediction is based on the principles of the heat equation, which governs how temperature and space evolve. The heat equation, which describes temperature evolution, is defined as in Equation 15.


$$\frac{\partial u}{\partial t}-k\frac{\partial^{2}u}{\partial x^{2}}=0$$ (15)

where ‘u’ represents temperature (PT in this case) and ‘k’ is the heat diffusion coefficient. The physics-driven loss is calculated using Equations 16 and 10 to calculate the data-driven loss. Now let us discuss the last Physics-based machine learning model used in the present work, based on the Schrödinger equation that describes the behavior of a quantum wave function (‘ψ’) and is defined as Equation 16.


$$\hat{H}\psi =i\hbar \frac{\partial \text{ψ}}{\partial t}$$ (16)

where ‘ψ’ represents the wave function, which is the model’s output, $\hat{H}$ is the Hamiltonian operator, which characterizes the energy of the quantum system, ‘i’ is the imaginary unit, and ‘ℏ’ is the reduced Planck’s constant. The physics-informed loss term, ‘Schrodinger loss,’ verifies that the model follows the Schrödinger equation as shown in Equation 17.


$$Schrödingerloss=\frac{1}{n}\sum_{i=1}^{n}\left({\left(\hat{H}\psi -i\hbar \frac{\partial \psi }{\partial t}\right)}^{2}\right)$$ (17)

**Fig 10:**
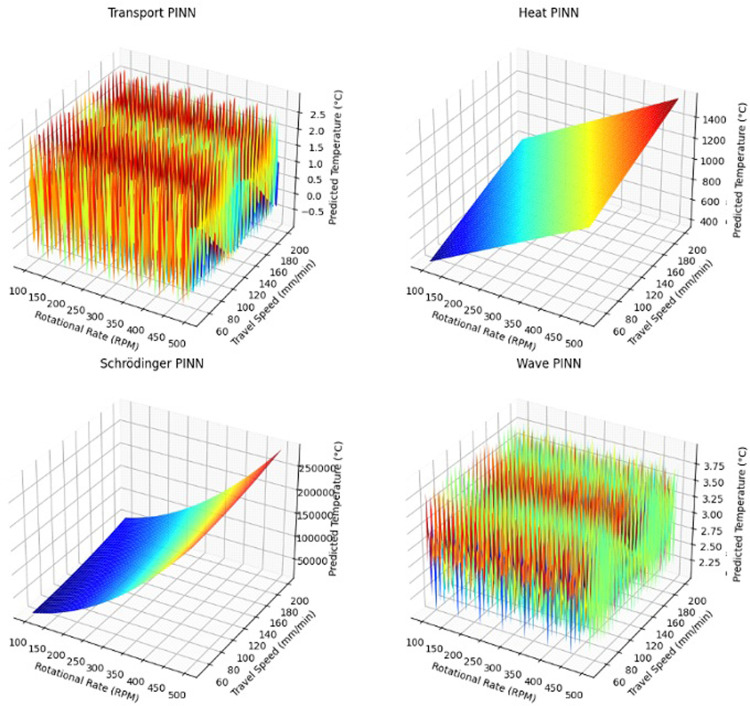
Surface plots of the used Physics machine learning models in the present work.

**Fig 11:**
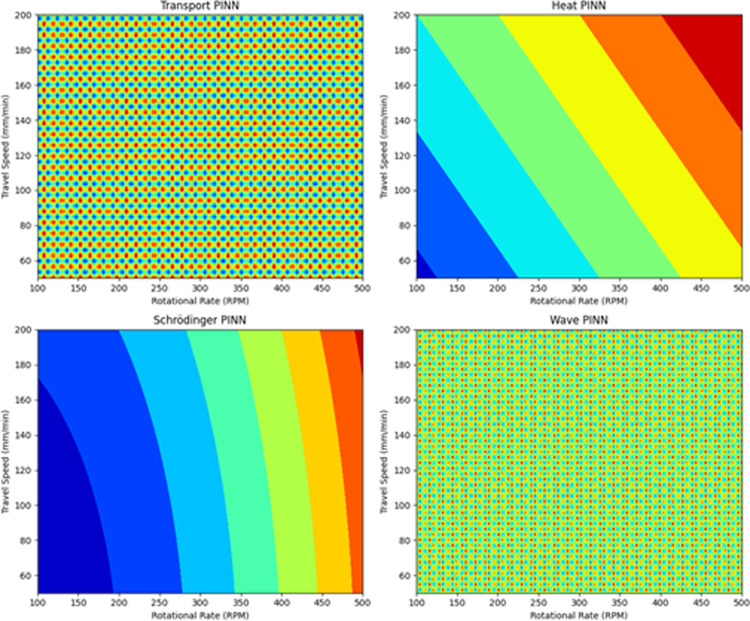
Contour Plot of Physics machine learning models used in the present work.

The surface and contour plots of the used physics-based machine learning model are shown in Figs 10 and 11. Table 2 shows the obtained metric features for physics-based machine learning algorithms.

**Table 2:** Results obtained from Physics based machine learning models.

Algorithms	RMSE Value	MAE value	Execution Time in seconds
Transport equation-based PINN model	64.6096	57.2337	83.5613
Wave equation-based PINN model	66.3619	55.9323	84.6350
Heat equation-based PINN model	65.2640	58.8696	84.7443
Schrödinger based PINN model	71.0866	61.4765	89.9683

The selection of specific equations (transport, wave, heat, and Schrödinger) is guided by their ability to accurately model the key physical phenomena in the AFSD process. Each equation addresses a different aspect of the process, from material flow and heat management to mechanical integrity and advanced material properties. Integrating these equations into physics-based machine learning algorithms helps in creating robust models that can predict and optimize the AFSD process, leading to improved material properties and process efficiency. Below is a detailed explanation of how these equations might be chosen and their relevance to AFSD:

Transport Equations: This equation is crucial for modeling the flow of material during the AFSD process. It helps in understanding how the material moves and redistributes during deposition.

Wave Equations: This is relevant for modeling the propagation of stress waves through the material during deposition. Understanding stress wave propagation is important for predicting the mechanical properties and potential residual stresses in the deposited material.

Heat Equations: The AFSD process involves significant heat generation due to friction and plastic deformation. The heat conduction equation models the temperature distribution within the material, which is crucial for predicting thermal gradients and cooling rates. Accurate modeling of heat generation at the tool-material interface and its dissipation through conduction, convection, and radiation is essential for controlling the microstructure and mechanical properties of the deposited material.

Schrödinger Equation: It used to model quantum effects in certain materials or nano-scale phenomena that influence the overall properties of the deposited material.

It is observed that the response surfaces are relatively smooth, with no sharp discontinuities or irregularities. This indicates that physics-informed neural networks model the system smoothly and capture coherent underlying dynamics. The peak temperature generally increases as rotational rate and travel speed increase, reflecting proper modeling of the process physics. There are some subtle differences between the models. For example, the heat equation model predicts slightly higher temperatures than the transport equation model under the same conditions. The contour plots also show smooth and evenly spaced contours, reaffirming continuous and well-behaved modeling by the physics-informed NNs. The contour lines are oriented diagonally, visualizing the coupled effect of rotational rate and travel speed on peak temperature.

The transport equation PINN model had the lowest RMSE of 64.6 and MAE of 57.2 among the physics-informed approaches. An RMSE of 64.6 in the context of AFSD suggests that, on average, the predicted values deviate from the actual values by approximately 64.6 units. For AFSD, this error could indicate deviations in material deposition thickness. An MAE of 57.2 indicates that, on average, the model’s predictions are off by about 57.2 units. In AFSD, this could mean an average deviation of 57.2 micrometers in deposition thickness. Heat equation PINN also produced low errors with RMSE of 65.2 and MAE of 58.9. This demonstrates that including physics knowledge about the underlying relationships dramatically improves predictive performance compared to pure data-driven supervised learning. Executing times for the PINN models ranged from 83 to 90 seconds, comparable to the slower supervised algorithms like CatBoost. However, their accuracy gains show the value of added physics-based constraints.

### 4.3. Supervised machine learning classification-based algorithms used for deposition quality prediction

**Fig 12:**
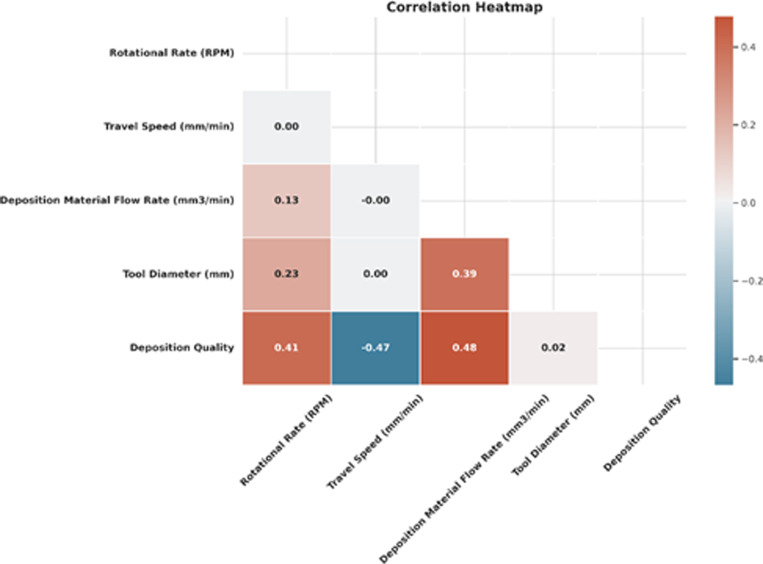
A correlation heatmap was obtained to classify the deposition quality in the present work.

Fig 12 shows the correlation heatmap for predicting deposition quality. We can see that the Deposition material rate has the strongest correlation with the target variable. The Rotational Rate has a weaker positive correlation. This suggests that deposition material rate is the most critical predictor of deposition quality.

**Fig 13:**
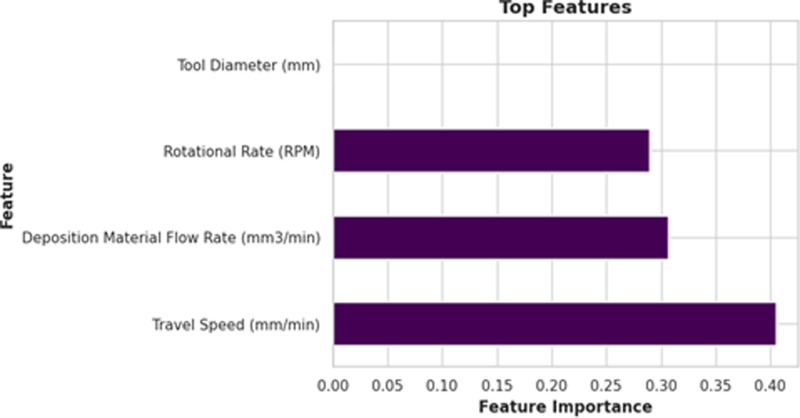
Feature importance plot obtained in the present work.

Fig 13 shows the feature importance plot. It is observed that the Travel Speed (mm/min) has the highest contribution towards the deposition quality while tool diameter (mm) has no contribution towards the deposition quality. So, before training the models, the tool diameter feature is dropped.

Logistic regression is a statistical method used for binary classification tasks in which the goal is to predict one of two possible classes (in present research work, “0” for poor deposition and “1” for good deposition) based on one or more input features (Rotational rate, Travel speed, and Deposition material flow rate). Logistic regression begins by solving a linear equation to calculate a linear combination of the input features. It can be described in the present research work with three input features, as shown in Equation 18.


$$z=\beta_{0}+\beta_{1}.\;RotationalRate+\beta_{2}.\;Travel\;Speed+\beta_{3}.\;DepositionMaterialFlowRate$$ (18)

where *z* is the linear combination of the input features, $\beta_{0}$ is the intercept term, $and\beta_{1}$, $\beta_{2}$, and $\beta_{3}$ are coefficients associated with each input feature. The linear combination z is then sent via a logistic function (a sigmoid function), which converts it to a number between 0 and 1. The logistic function is described in Equation 19.


$$\sigma \left(z\right)=\frac{1}{1+e^{-z}}$$ (19)

where *σ*(*z*) represents the probability that the target variable belongs to class “1” (good deposition), because the logistic function assures that the anticipated values lie inside the range [0, 1], it is appropriate for modeling probabilities. Following the calculation of *σ* (z), a threshold (typically 0.5) is used to establish the final anticipated class label. If *σ* (z) is greater than or equal to 0.5, the occurrence is categorized as “1” (excellent deposition), otherwise as “0” (poor deposition). In K-Nearest Neighbours, the prediction step determines the distance between the new data point (query point) and all data points in the training dataset. The Euclidean distance, represented as Equation 20, is widely employed.


$$EucledianDistance\left(X,X_{i}\right)=\sqrt{\sum_{j=1}^{n}{\left(X-X_{i}\right)}^{2}}$$ (20)

**Fig 14:**
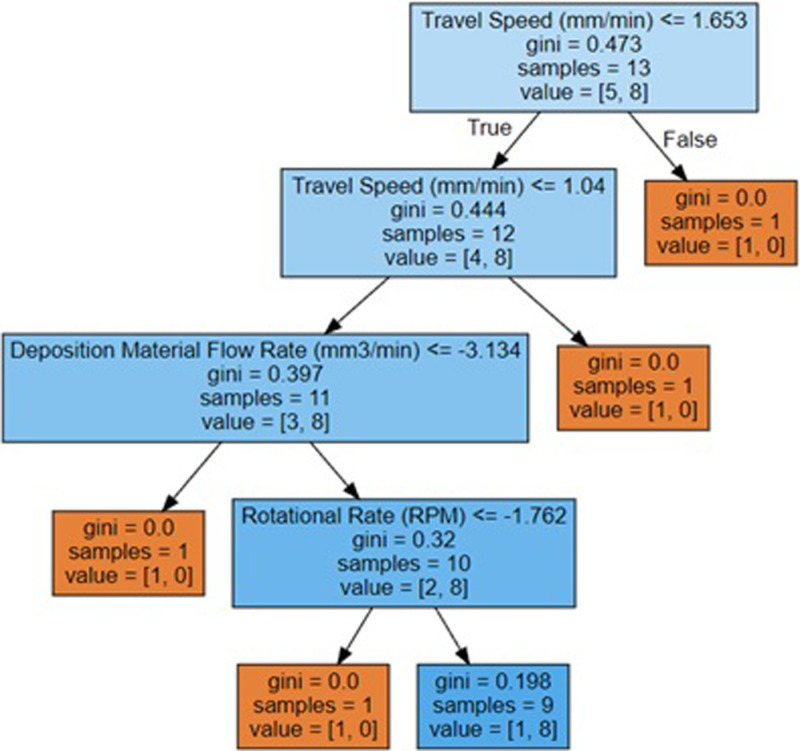
Decision tree plot obtained for classification-based model.


*X* is the feature vector of the query point, $X_{i}$ is the feature vector of the *i*-th data point in the training dataset, and *n* is the number of features. When using K-NN for binary classification (predicting deposition quality as “0” for bad and “1” for good), the algorithm counts the number of neighbors from each class among the K nearest neighbors. It selects the class label that appears the most frequently among the query point’s K neighbors as the anticipated class.Support Vector Classification seeks a hyperplane (a decision boundary) that best separates data points from distinct classes while maximizing margin. The data points nearest to the hyperplane are known as support vectors, and they are critical in establishing the margin and generating the decision boundary. The goal is to create a hyperplane that fulfills the following equation for all support vectors Xi, as shown in Equation 21.


$$y_{i}\left(w.\;X_{i}+b\right)\geq 1$$ (21)

where $y_{i}$ is the class label of the i-th data point ($y_{i}=1$ for the positive class and $y_{i}=0$ for the negative class), w is the weight vector (average to the hyperplane), $X_{i}$ is the feature vector of the *i*-th data point, and b is the bias term. Data is frequently not perfectly separated by a hyperplane in real-world circumstances. SVC offers the concept of a “soft margin” in order to allow for occasional misclassifications while still increasing margins. This is accomplished by including a parameter C that regulates the trade-off between margin maximization and misclassification tolerance. On the other hand, the mathematical formulation of the parameter update in Stochastic Gradient Descent (SGD) is shown in Equation 22.


$$\theta =\theta -\alpha .\nabla J\left(\theta \right)$$ (22)

where *θ* represents the model parameters, *α* is the learning rate, and $\nabla J\left(\theta \right)$ is the gradient of the cost function concerning θ. At each iteration, SGD introduces randomization by selecting random data points (or mini-batches). This randomization aids in avoiding local minima and may result in faster convergence. While SGD converges to the cost function’s minimum, it may oscillate about it. However, it frequently arrives at a “good enough” solution faster than classical gradient descent. The mathematical framework of Decision Tree Classification consists mainly of calculating impurity measurements, shown in Equation 23, and picking the appropriate feature to partition the data, as shown in Fig 14.

$$Impurity=\sum_{i=1}^{n}p\left(i\right).\left(1-p\left(i\right)\right)$$ (23)

where n is the number of classes and $p\left(i\right)$ is the proportion of data points that belong to class i.

**Fig 15:**
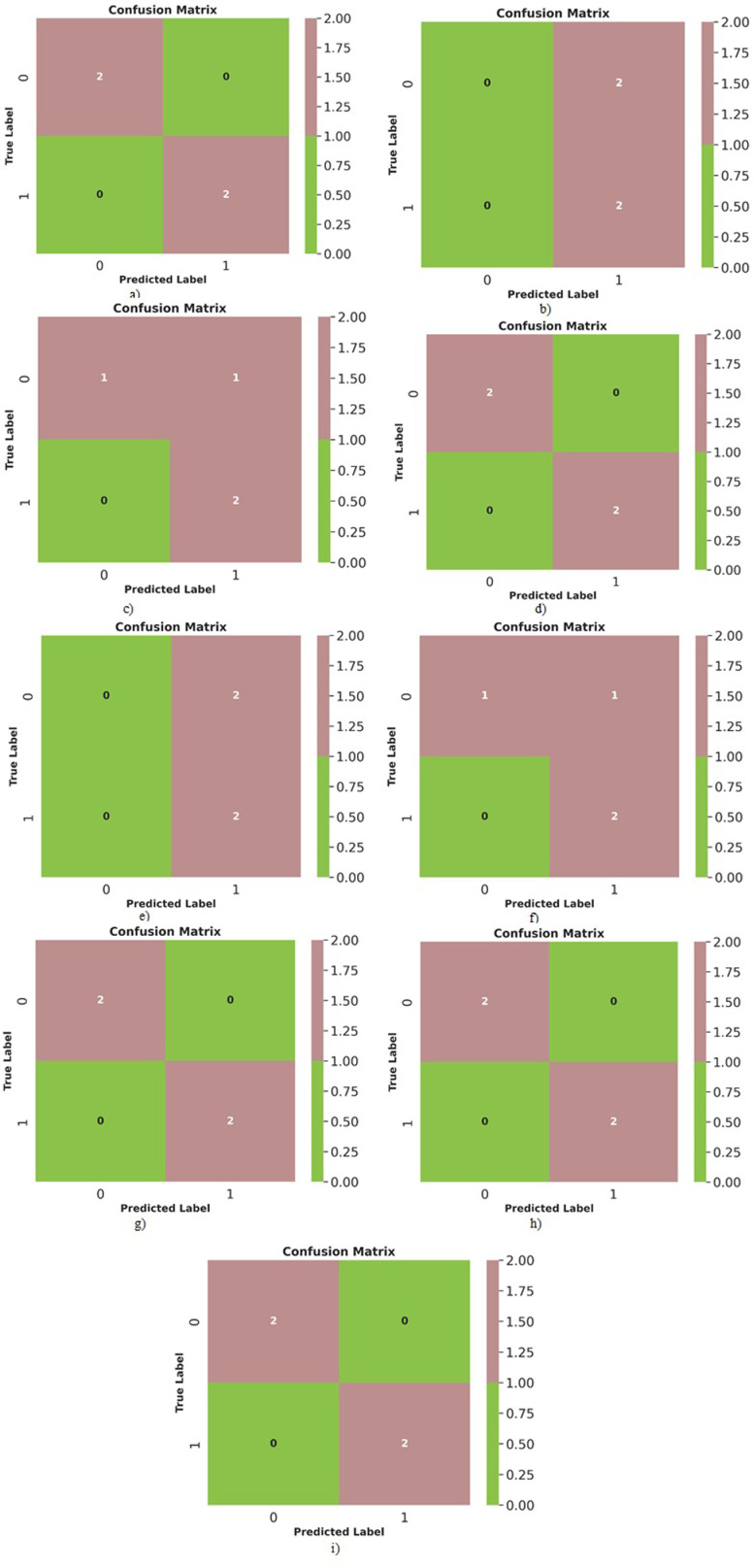
Confusion matrix plots obtained for a) Logistic, b) K-Nearest Neighbours, c) Support Vector, d) Stochastic Gradient Descent, e) Decision Tree, f) Random Forest, g) AdaBoost, h) Gradient Boosting and i) Stochastic Gradient Boosting algorithms.

**Fig 16:**
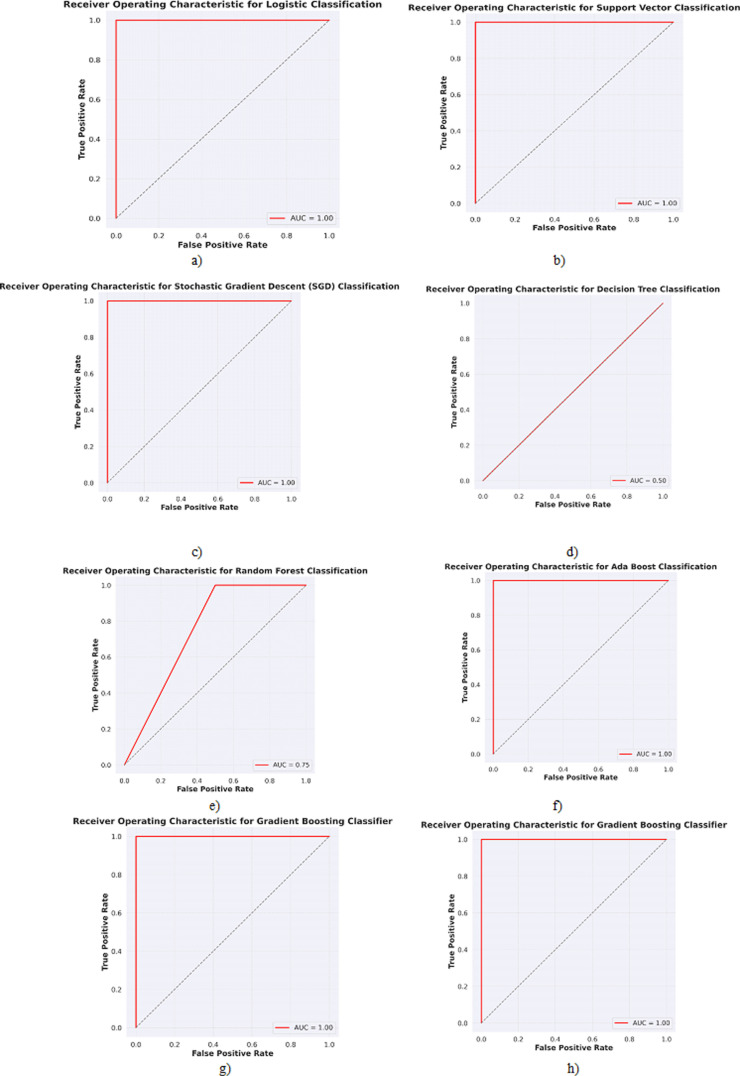
ROC plots were obtained for a) Logistic, b) K-Nearest Neighbours, c) Support Vector, d) Stochastic Gradient Descent, e) Decision Tree, f) Random Forest, g) AdaBoost, h) Gradient Boosting and i) Stochastic Gradient Boosting algorithms.

Similarly, Random Forest’s mathematical formulation consists mainly of calculating impurity measurements, picking the optimal feature to partition the data, and merging the predictions from separate trees. Bootstrapped samples and random feature subsets are used to reduce overfitting. Random Forest Classification is a versatile and durable algorithm noted for its excellent accuracy and resistance to overfitting. Gradient Boosting and Stochastic Gradient Boosting are powerful strategies for creating highly accurate models. They are frequently utilized in machine learning contests as well as real-world applications. The primary distinction is how data is subsampled and how the learning rate is modified. Because of its stochastic nature, stochastic gradient boosting is often preferred when dealing with big datasets or when computer resources are restricted. Table 3 shows the obtained metric features for the classification-based algorithms. Fig 15 shows the confusion matrix plot for the implemented classification-based algorithms, while Fig 16 shows the plot for Receiver Operating characteristics plots for each implemented algorithm.

**Table 3:** Results obtained from the supervised classification-based machine learning algorithms to predict deposition quality.

Algorithms	Execution Time in seconds	Training data accuracy	Test data accuracy	Overall F1-Score	ROC-AUC Score
Logistic	0.0203	0.9230	1.0	1.0	1.0
K-Nearest Neighbours	0.0060	0.6153	0.5	0.5	1.0
Support Vector	0.5860	1.0	0.75	0.75	1.0
Stochastic Gradient Descent	4.4190	1.0	1.0	1.0	1.0
Decision Tree	0.0012	0.9230	0.5	0.5	0.5
Random Forest	0.3400	0.9230	0.75	0.75	0.75
AdaBoost	0.0100	1.0	1.0	1.0	1.0
Gradient Boosting	0.1300	1.0	1.0	1.0	1.0

Based on the results in Table 3, Figs 15, and 16 for the classification algorithms predicting deposition quality, Logistic regression achieved perfect accuracy on both the training and test data with a ROC-AUC score of 1.0. The confusion matrix correctly classifies all excellent and poor depositions. False positives and false negatives have critical implications in the context of AFSD quality prediction. Minimizing these errors is essential for maintaining production efficiency, ensuring product reliability, and optimizing costs. By carefully balancing these errors through strategic model tuning and validation, manufacturers can improve the overall performance and reliability of their machine learning models, leading to better outcomes in the AFSD process. This indicates that logistic regression fits the data very well. K-nearest neighbors had low accuracy on the test data (50%) and training data (61.53%). The confusion matrix shows it needed help differentiating between excellent and poor depositions. However, it still achieved a ROC-AUC score of 1.0, suggesting potential with tuning. The support vector classifier had 100% training accuracy but only 75% test accuracy. The confusion matrix shows it incorrectly classified some poor depositions as good. Its ROC-AUC score of 1.0 indicates good discrimination when tuning the decision threshold. Stochastic gradient descent matched logistic regression with 100% accuracy on both training and testing. The confusion matrix shows perfect classification. The ROC-AUC of 1.0 also indicates excellent performance. Decision tree had high training accuracy (92.3%) but low test accuracy (50%). The confusion matrix reveals overfitting as it struggled with the unseen test data. The poor ROC-AUC score of 0.5 confirms this. Random forest improved upon decision tree with 75% test accuracy and ROC-AUC of 0.75. The confusion matrix correctly classified more good and poor depositions than decision trees. AdaBoost, gradient boosting, and stochastic gradient boosting all achieved 100% training and testing accuracy with ROC-AUC of 1.0. Their confusion matrices show perfect classification of all samples. K-Nearest Neighbors and Decision Tree models may underperform due to sensitivity to noise, and high dimensionality. By implementing strategies such as cross-validation, hyperparameter tuning, feature scaling, and ensemble methods, the predictive capabilities of these models can be significantly improved. Understanding and addressing these factors will enhance the models’ accuracy and robustness, making them more suitable for real-world deployment in predicting deposition quality in AFSD.

## 5. Conclusion

This work demonstrated a novel integration of supervised machine learning and physics-informed neural networks to model peak temperature and deposition quality in additive friction stir deposition processes. Across several statistical measures, ensemble methods like gradient boosting and CatBoost proved the most effective for regression-based peak temperature prediction within the supervised learning models. However, physics-informed models leveraging governing transport, wave, and heat equations significantly outperformed the data-driven approaches, achieving the lowest errors by incorporating physical constraints. Techniques like logistic regression and stochastic gradient descent for classifying deposition quality delivered robust accuracy. The dual framework combining statistical and physics-based modeling provides unique insights into correlating process parameters to thermal profiles and deposition performance in AFSD. By elucidating these relationships, the integrated approach facilitates the optimized design of AFSD processes for tailored microstructural properties. This work highlights the merits of synergistically blending data-driven and physics-based techniques to uncover engineering design principles linking manufacturing processes to materials structure and properties.

While the proposed machine learning approaches show promise for improving deposition quality prediction in additive friction stir deposition, there are several limitations and challenges that need to be addressed. By considering the quality and quantity of data, model interpretability, computational complexity, and generalization to different conditions, the robustness and reliability of these models can be enhanced. Additionally, extending the scope to other additive manufacturing processes, integrating physical models, addressing material variability, and ensuring scalability and ethical considerations are key areas for future research and development. Addressing these factors will help in the broader application and acceptance of machine learning techniques in additive manufacturing.

## Acknowledgment

The authors extend their appreciation to King Saud University for funding this work through the Researchers Supporting Project number (RSP2025R164), King Saud University, Riyadh, Saudi Arabia.

## References

[1] Maleki E, Bagherifard S, Bandini M, Guagliano M. Surface post-treatments for metal additive manufacturing: Progress, challenges, and opportunities. Additive Manufacturing. 2021;37:101619. doi: 10.1016/j.addma.2020.101619

[2] Xu Z, La Mendola I, Razavi SMJ, Bagherifard S. Additive manufactured triply periodic minimal surface lattice structures with modulated hybrid topology. Engineering Structures. 2023;289:116249.

[3] Maleki E, Bagherifard S, Heydari Astaraee A, Sgarbazzini S, Bandini M, Guagliano M. Application of gradient severe shot peening as a novel mechanical surface treatment on fatigue behavior of additively manufactured AlSi10Mg. Materials Science and Engineering: A. 2023;881:145397. doi: 10.1016/j.msea.2023.145397

[4] Tamburrino F, Graziosi S, Bordegoni M. The design process of additively manufactured mesoscale lattice structures: a review. Journal of Computing and Information Science in Engineering. 2018;18(4):040801.

[5] Bregoli C, Buccino F, Picca F, Bagherifard S, Biffi CA, Tuissi A, et al. Additive manufacturing of AISI 316L specimens with distributed inner bone-type cavities: processability and characterization. IOP Conference Series: Materials Science and Engineering. 2023;1275(1):012001.

[6] Agrawal P, Haridas RS, Yadav S, Thapliyal S, Gaddam S, Verma R, et al. Processing-structure-property correlation in additive friction stir deposited Ti-6Al-4V alloy from recycled metal chips. Additive Manufacturing. 2021;47:102259.

[7] Agrawal P, Haridas RS, Mishra RS. Deformation-based additive manufacturing of a metastable high entropy alloy via Additive friction stir deposition. Additive Manufacturing. 2022;60:103282. doi: 10.1016/j.addma.2022.103282

[8] Martin LP, Luccitti A, Walluk M. Repair of aluminum 6061 plates by additive friction stir deposition. The International Journal of Advanced Manufacturing Technology. 2022:1-15.

[9] Khodabakhshi F, Gerlich AP. Potentials and strategies of solid-state additive friction-stir manufacturing technology: A critical review. Journal of Manufacturing Processes. 2018;36:77–92.

[10] Yu HZ, Hahn GD. Potential and challenges for large-scale near-net-shaping of 7xxx aerospace grade aluminum via additive friction stir deposition. Materials Letters: X. 2023;19:100217. doi: 10.1016/j.mlblux.2023.100217

[11] Wu B, Peng Y, Tang H, Meng C, Zhong Y, Zhang F, et al. Improving grain structure and dispersoid distribution of nanodiamond reinforced AA6061 matrix composite coatings via high-speed additive friction stir deposition. Journal of Materials Processing Technology. 2023;317:117983. doi: 10.1016/j.jmatprotec.2023.117983

[12] Li Y, Yang B, Zhang M, Wang H, Gong W, Lai R, et al. The corrosion behavior and mechanical properties of 5083 Al-Mg alloy manufactured by additive friction stir deposition. Corrosion Science. 2023;213:110972.

[13] Mukhopadhyay A, Saha P, Singh PK, Verma M. Development and analysis of a powder bed friction stir (PBFS) additive manufacturing process for aluminum alloys: A study on friction-stirring pitch (ω/v) and print location. Additive Manufacturing. 2023;72:103618.

[14] El-Sayed Seleman MM, Ataya S, Ahmed MMZ, Hassan AMM, Latief FH, Hajlaoui K, et al. The Additive Manufacturing of Aluminum Matrix Nano Al2O3 Composites Produced via Friction Stir Deposition Using Different Initial Material Conditions. Materials (Basel). 2022;15(8):2926. doi: 10.3390/ma15082926 35454620 PMC9029182

[15] Yoder JK, Hahn GD, Zhao N, Brennan RE, Cho K, Hang ZY. Additive friction stir deposition-enabled upcycling of automotive cast aluminum chips. Additive Manufacturing Letters. 2023;4:100108.

[16] Robinson TW, Williams MB, Rao HM, Kinser RP, Allison PG, Jordon JB. Microstructural and mechanical properties of a solid-state additive manufactured magnesium alloy. Journal of Manufacturing Science and Engineering. 2022;144(6):061013.

[17] Choudhury S, Acharya U, Roy J, Roy BS. Recent progress in solid-state additive manufacturing technique: Friction stir additive manufacturing. Proceedings of the Institution of Mechanical Engineers, Part E: Journal of Process Mechanical Engineering. 2022;237(2):467–91. doi: 10.1177/09544089221107755

[18] Ralls AM, Menezes PL. Understanding the tribal-corrosion mechanisms of friction stir processed steel deposited by high-pressure deposition additive manufacturing process. The International Journal of Advanced Manufacturing Technology. 2023;128(1):823–43.

[19] Liu L, Xu W, Zhao Y, Lin Z, Liu Z, Dong Y, et al. Tailoring the microstructure and mechanical properties of wire and arc additive manufactured Al-Mg alloy via interlayer friction stir processing. Journal of Materials Research and Technology. 2023.

[20] Sharma S, Krishna KM, Radhakrishnan M, Pantawane MV, Patil SM, Joshi SS, et al. A pseudo thermo-mechanical model linking process parameters to microstructural evolution in multilayer additive friction stir deposition of magnesium alloy. Materials & Design. 2022;224:111412.

[21] Joshi SS, Patil SM, Mazumder S, Sharma S, Riley DA, Dowden S, et al. Additive friction stir deposition of AZ31B magnesium alloy. Journal of Magnesium and Alloys. 2022;10(9):2404–20. doi: 10.1016/j.jma.2022.03.011

[22] Gotawala N, Hang ZY. Material flow path and extreme thermomechanical processing history during additive friction stir deposition. Journal of Manufacturing Processes. 2023;101:114–27.

[23] Chaudhary B, Jain NK, Murugesan J. Experimental investigation and parametric optimization of friction stir powder additive manufacturing process for aerospace-grade Al alloy. The International Journal of Advanced Manufacturing Technology. 2022;123(1–2):603–25.

[24] Chaudhary B, Jain NK, Murugesan J. Development of friction stir powder deposition process for repairing aerospace-grade aluminum alloys. CIRP Journal of Manufacturing Science and Technology. 2022;38:252–67.

